# Optimizing *Cucumis sativus* seedling vigor: the role of pistachio wood vinegar and date palm compost in nutrient mobilization

**DOI:** 10.1186/s12870-024-05128-y

**Published:** 2024-05-16

**Authors:** Sediqeh Afsharipour, Abdolmajid Mirzaalian Dastjerdi, Azam Seyedi

**Affiliations:** 1 Department of Agricultural Engineering, University of Hormozgan, Bandar Abbas, Iran; 2 Department of Horticultural Science, Faculty of Agriculture, University of Jiroft, Jiroft, Iran

**Keywords:** Auxin, Cucumber seedling, Culture media, Pyroligneous acid, Root architecture

## Abstract

**Background:**

The goal of this research is to enhance the quality of cucumber seedlings grown in greenhouses by experimenting with various soilless culture mediums (CMs) and the application of pistachio wood vinegar (WV). The experimental setup was designed as a factorial experiment within a randomized complete block design (RCBD), in greenhouse conditions featuring three replications to assess the effects of different culture media (CMs) and concentrations of pistachio wood vinegar (WV) on cucumber seedling growth. Cucumber seeds were planted in three CMs: coco peat-peat moss, coco peat-vermicompost, and date palm compost-vermicompost mixed in a 75:25 volume-to-volume ratio. These were then treated with pistachio WV at concentrations of 0, 0.5, and 1%, applied four times during irrigation following the emergence of the third leaf.

**Results:**

The study revealed that treating seedlings with 0.5% WV in the date palm compost-vermicompost CM significantly enhanced various growth parameters. Specifically, it resulted in a 90% increase in shoot fresh mass, a 59% increase in shoot dry mass, an 11% increase in root fresh mass, a 36% increase in root dry mass, a 65% increase in shoot length, a 62% increase in leaf area, a 25% increase in stem diameter, a 41% increase in relative water content (RWC), and a 6% improvement in membrane stability index (MSI), all in comparison to untreated seedlings grown in coco peat-peat moss CM. Furthermore, chlorophyll a, b, total chlorophyll, and carotenoid levels were 2.3, 2.7, 2.6, and 2.7 times higher, respectively, in seedlings treated with 0.5% WV and grown in the date palm compost-vermicompost CM, compared to those treated with the same concentration of WV but grown in coco peat-peat moss CM. Additionally, the Fv/Fm ratio saw a 52% increase. When plant nutrition was enhanced with the date palm compost-vermicompost CM and 1% WV, auxin content rose by 130% compared to seedlings grown in coco peat-peat moss CM and treated with 0.5% WV.

**Conclusions:**

The study demonstrates that using 0.5% WV in conjunction with date palm compost-vermicompost CM significantly betters the quality of cucumber seedlings, outperforming other treatment combinations.

## Background

The degradation of soil quality in arid and semi-arid regions is a growing concern due to factors such as organic matter depletion, overuse of chemical fertilizers, salinization, desertification, water scarcity, and increased erosion rates. Addressing these challenges necessitates the adoption of soilless culture techniques and the development of innovative culture media (CMs) characterized by specific physicochemical attributes. Cucumber (*Cucumis sativus* L.) emerges as a crop of global significance, celebrated for its commercial value and myriad health benefits [1, 2]. The world witnessed a staggering annual cucumber production of approximately 91.3 million tons in 2020, with Iran ranking as the fourth largest producer, yielding 1.2 million tons annually, predominantly in the southern Kerman province, a hotspot for greenhouse vegetable cultivation [3].

Soilless cultivation has gained attention due to its efficiency in managing growth medium, water and nutrients, salinity, microorganisms and product quality, thereby offering a viable alternative to traditional soil-based cultivation [4, 5]. These systems utilize a variety of materials as CMs, each possessing unique properties essential for supporting plant growth, including adequate aeration, efficient drainage, high water retention, and substantial cation exchange capacity, and without harmful effects on plants [5, 6]. The selection of an appropriate CM is pivotal, with options encompassing a blend of organic substances like peat moss, wood residues, coconut fibers, sugarcane pulp, decomposed leaves, rice husks, and mineral components such as vermiculite, perlite, rock wool, polyester foam, and sand, each contributing distinct advantages to plant development [6, 7].

Wood vinegar (WV), a by-product of wood pyrolysis, stands out as an important organic compound in agriculture and has a range of benefits including plant growth stimulation, high acidity, numerous volatile compounds, antioxidant, antimicrobial, and anti-inflammatory properties. The antifungal properties of WV provide soil improvement, seed germination, root growth, and pest control. WV consists of 80–90% water and a major component of acetic acid, along with about 200 other organic compounds such as acids, alcohols, phenols, esters, and aldehydes [8–11]. It has long been used in agriculture and forestry as a fertilizer, plant growth-stimulating agent, herbicide, and soil amendment, in animal husbandry as a feed additive, in environmental protection for the treatment of aluminum ore residues, and production of acetic acid or snow-melting agent [12]. In this study, we selected a locally produced wood vinegar obtained from biochar production using pistachio wood pruning waste as a raw material. Initially, wood vinegar was chemically investigated through gas chromatography-mass spectrometry (GC-MS). After that, we investigated the effectiveness of wood vinegar as a growth stimulant on the quality of cucumber seedlings. The specific hypotheses tested in this study were as; 1-Wood vinegar is acidic and rich in biologically active compounds, which leads to an increase in the quality of cucumber seedlings. 2-Wood vinegar shows concentration-dependent effects on cucumber seedling quality, exhibits phytotoxicity at high concentrations, and is biostimulant when significantly diluted. Also, in this study, we used locally produced culture medium (palm compost) in combination with other culture mediums. At first, palm compost was compared with other cultivation substrates after checking its chemical composition. So, we investigated the efficiency of palm compost as a local cultivation substrate on the quality of cucumber seedlings. Given the critical role of CM and nutrition in seedling production, this study aims to explore the efficacy of various soilless CMs combined with pistachio WV in improving the quality of greenhouse-grown cucumber seedlings.

## Results and discussion

### Evaluation of root architecture

The study’s results, as detailed in Table 1, reveal that the combination of culture medium (CM) and wood vinegar (WV) significantly influences root morphology, impacting parameters such as total root length, root volume, number of lateral roots, and root width, among others. This interaction shows the roots’ critical role in environmental perception and subsequent growth adaptation of the plant’s shoot system. Roots, by detecting environmental signals first, initiate responses that shape plant growth. The observed changes in root characteristics, such as increased root length and lateral root formation, suggest enhanced water and nutrient uptake capabilities, essential for plant health and productivity. Moreover, our study highlights how these root traits can serve as indicators of root system quality and efficiency. The interaction between CM and WV presents a promising avenue for optimizing root development. By manipulating root architecture through these means, plants can be better equipped to absorb essential resources, ultimately supporting improved growth and resilience. This insight opens up avenues for agricultural practices aimed at enhancing plant development through targeted adjustments in the growing medium and amendments.

**Table 1:** Variance analysis for the effect of culture medium and wood vinegar on root architecture of cucumber seedlings

Variation sources	DF	Total root length	Root volume	Lateral roots number	Root width
Culture medium	2	24536058.2^**^	235247.81^**^	501.11^**^	2517.55^**^
Wood vinegar	2	787457.68^*^	6206.88^ns^	5.77^*^	7761.94^**^
CM × WV	4	1148722.63^**^	28573.41^**^	39.23^**^	19963.32^**^
C.V.	-	6.43	7.50	4.69	6.59
**Variation sources**	**DF**	**Root width/ depth**	**Root perimeter**	**Root area**	**Specific root length**
Culture medium	2	0.065^*^	73510983.6^**^	1572671.31^**^	2.55^**^
Wood vinegar	2	0.093^**^	1020049.1^*^	71920.91^*^	1.51^*^
CM × WV	4	0.281^**^	2847507.6^**^	120179.17^**^	1.09^*^
C.V.	-	9.96	4.78	7.19	5.79

^*^ and ^**^ indicate the presence of a significant difference at the 0.01 and 0.05 levels, respectively, and ^ns^ indicates the absence of a significant difference

### Total root length and root volume

**Fig. 1:**
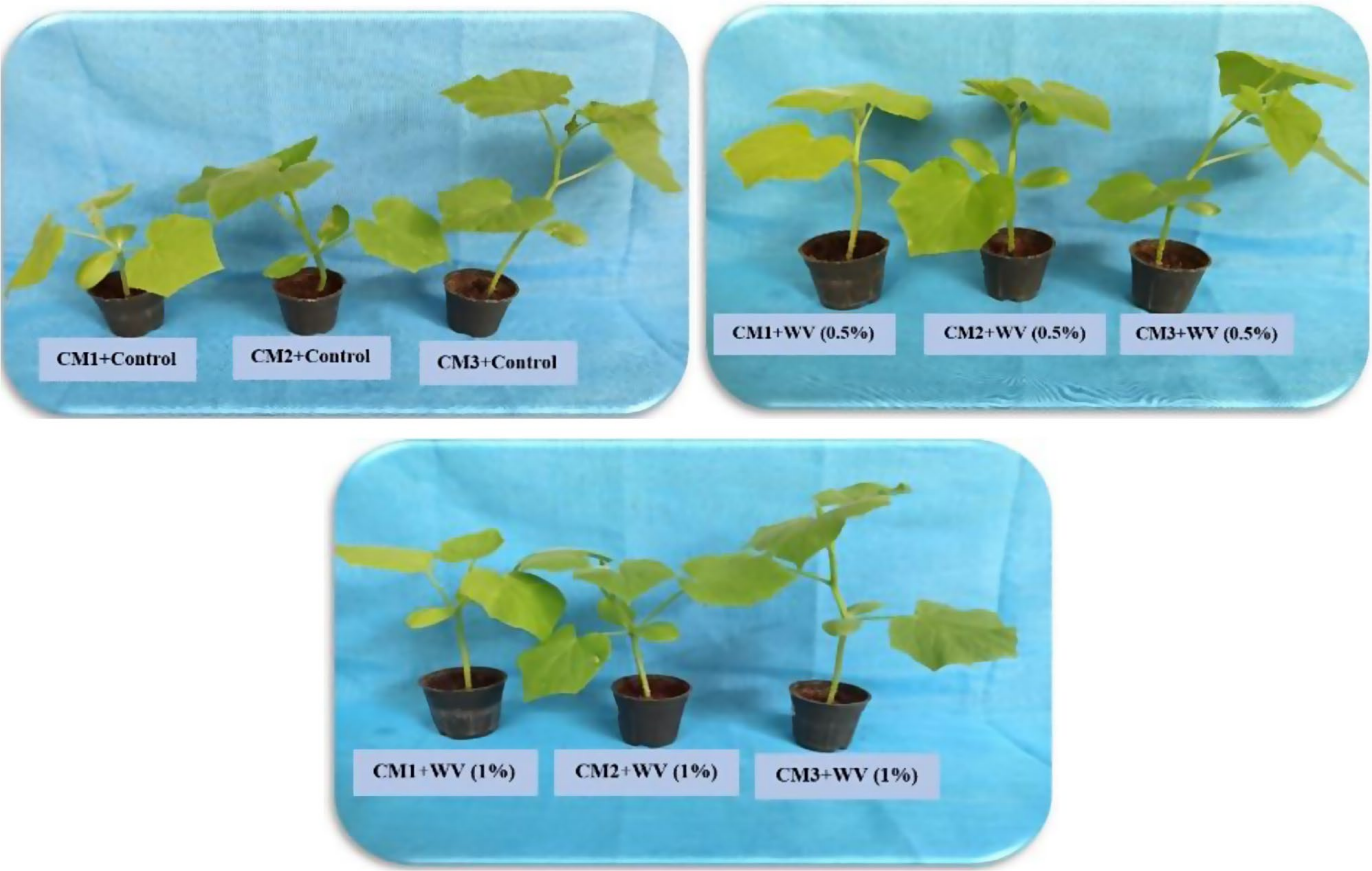
CM1: Coco peat-peat moss, CM2: Coco peat-vermicompost, CM3: Date palm compost-vermicompost, WV, wood vinegar

In an investigation delineated within Table 2, seedlings subjected to 1% wood vinegar (WV) treatment within a coco peat-peat moss culture medium (CM) demonstrated significantly enhanced root development, exhibiting a total root length of 8360.2 cm and a root volume of 917.02 cm^− 3^. Notably, this treatment facilitated an 80% increase in root volume relative to seedlings cultivated in a date palm compost-vermicompost CM without WV application. Furthermore, Fig. 1 elucidates a statistically significant and positive correlation between total root length and various root metrics including particular root length (PRL), root volume (RV), and root perimeter (RP), as well as between RV and root area (RA). Pistachio wood vinegar, is an acidic compound that also contains phenolic compounds such as; 2-methoxy-4-methylphenol, phenol, 2,6-dimethoxyphenol, guaiacol, p-octylphenol (Table 3), can improve the root grow. According to the results of previous researchers, the organic compounds of wood vinegar such as acids, phenols and ketones, by affecting the interactions within the cell walls, lead to acid secretions from the roots, strengthening the rhizosphere, and improvement of root growth. Our results are consistent with the improvement of traits related to root architecture [13, 14]. Moreover, WV has been implicated in enhancing plant growth by augmenting chlorophyll content, photosynthesis rates, and root strength [15]. The significant alteration in plant root architecture, as influenced by environmental conditions and soil nutrients, plays a pivotal role in enhancing plant development, thereby exerting a profound effect on overall plant performance and yield [16]. The extension of the main root is instrumental for the deep-soil extraction of water and nutrients. Furthermore, lateral roots, constituting a critical component of the root system, significantly influence the overall surface area and activity level of the root system, thereby impacting plant growth and development [14].

**Table 2:** Mean comparison of the culture medium and wood vinegar on root architecture of cucumber seedlings

Treatments		Total root length (cm)	Root volume (cm^3^)	Lateral roots number	Root width (cm)
Culture media	Wood vinegar				
Coco peat - peat moss	0	8076.07^a^	864.13^ab^	32.00^b^	262.41^b^
	0.5%	8082.49^a^	773.13^bc^	27.44^c^	231.87^c^
	1%	8360.22^a^	917.02^a^	37.00^a^	322.79^a^
Coco peat - vermicompost	0	5253.72^c^	647.11^d^	25.52^cd^	252.66^bc^
	0.5%	7007.99^b^	867.82^ab^	27.89^c^	318.50^a^
	1%	6743.89^b^	664.56^cd^	24.22^d^	155.25^d^
date palm compost -vermicompost	0	5080.26^c^	507.26^e^	16.22^e^	173.29^d^
	0.5%	5053.71^c^	523.58^e^	18.78^e^	311.33^a^
	1%	4499.98^c^	561.19^de^	16.85^e^	324.38^a^
**Culture media**	**Wood vinegar**	**Root width/ depth**	**Root perimeter**	**Root area (cm** ^**2**^ **)**	**Specific root length (cm)**
Coco peat - peat moss	0	1.17^ab^	13031.83^bc^	1986.29^a^	9.26^b^
	0.5%	1.03^b^	13790.66^ab^	2015.47^a^	10.73^a^
	1%	1.32^a^	14342.79^a^	2145.97^a^	9.24^b^
Coco peat - vermicompost	0	1.06^b^	10134.19^e^	1429.66^bc^	8.75^bc^
	0.5%	1.29^a^	12206.34^cd^	1993.08^a^	8.71^bc^
	1%	0.67^c^	11495.11^d^	1519.02^b^	9.04^b^
date palm compost -vermicompost	0	0.75^c^	8851.69^f^	1289.16^bcd^	9.11^b^
	0.5%	1.27^a^	8040.65^fg^	1204.91^cd^	9.28^b^
	1%	1.32^a^	7182.75^g^	1146.14^d^	8.02^c^

Different letters in a column indicate significant differences at *p* ≤ 0.05 among the growing mediums, based on Duncan’s multiple-range test

**Table 3:** ANOVA results of different culture media (CM) and wood vinegar (WV) concentrations on growth parameters, RWC, and MSI of cucumber seedlings

Variation sources		Fresh mass			Dry mass					
	DF	Root	Shoot	shoot/root	Root		Shoot		shoot/root	
CM	2	0.33^**^	52.39^**^	4.71^**^	0.004^**^		0.26^**^		1.84^**^	
WV	2	0.045^*^	0.98^**^	0.16^*^	0.0004^*^		0.002^*^		0.43^**^	
CM*WV	4	0.10^**^	1.27^**^	0.24^**^	0.0002^*^		0.003^**^		0.25^*^	
C.V.	-	4.11	2.68	5.12	5.81		2.83		3.93	
**Variation sources**	**DF**	**Root length**	**Shoot length**	**Stem diameter**	**Stem sturdiness quotient**	**Leaf area**		**RWC**		**MSI**
CM	2	34.29^**^	140.38^**^	1.55^**^	2.13^**^	523.88^**^		734.50^**^		19.15^**^
WV	2	5.47^**^	0.73^ns^	0.11^ns^	0.04^ns^	9.24^**^		29.39^ns^		16.91^**^
CM*WV	4	4.58^**^	26.37^**^	0.34^**^	0.32^**^	6.61^*^		32.31^*^		33.56^**^
C.V.	-	7.42	2.73	4.70	3.36	3.25		3.81		1.91

* and ** indicate the presence of a significant difference at the 0.01 and 0.05 levels, respectively, and ns indicates the absence of a significant difference

### Lateral root number

The comparative analysis of seedlings grown in different culture mediums (CMs), as presented in Table 2, reveals a stark contrast in lateral root formation contingent upon the medium and treatment applied. Specifically, seedlings cultivated in a date palm compost-vermicompost CM, devoid of wood vinegar (WV) treatment, exhibited a significantly reduced number of lateral roots, averaging 16.2, which is approximately 2.3 times less than those grown in a coco peat-peat moss CM supplemented with 1% WV. This disparity underscores the crucial role of lateral roots in the overall architecture and functional capacity of the root system, influencing the specific surface, activity, and volume of the root. Lateral roots are integral to enhancing the root system’s total area and volume, thereby facilitating improved water and nutrient absorption, and increasing the root system’s activity. Lu et al. [14] have elucidated that the number of lateral roots and the uniformity of the root system are positively influenced by low concentrations of WV, highlighting the nuanced impact of WV concentration on root development. Conversely, higher concentrations of WV were observed to detrimentally affect these parameters, suggesting an optimal threshold for WV application that maximizes root growth and system uniformity. Furthermore, the selection of an appropriate CM is pivotal for root development, as it significantly influences oxygen transport to the roots and facilitates gas exchange between the roots and the atmosphere, crucial for optimal plant growth and development [17, 18]. This research collectively indicates that the strategic incorporation of WV in specific concentrations, alongside the choice of a suitable CM, can profoundly impact the development and functionality of the root system, thereby influencing overall plant health and productivity.

### Root width and the ratio of root width/depth

The analysis presented in Fig. 1 reveals a notable and positive correlation between root width (RW) and the ratio of root width to depth (RWD), suggesting that as root width increases, the proportionate depth adjustment follows to maintain an optimal structural balance. Interestingly, seedlings cultivated in coco peat-vermicompost CM and treated with 1% wood vinegar (WV) exhibited the smallest root width (155.3 cm) and the lowest root width/depth ratio (0.67). In contrast, seedlings that received 1% WV treatment in a date palm compost-vermicompost CM demonstrated the largest root width (324.4 cm), with the root width/depth ratio in these seedlings showing a 97% increase compared to those grown in the coco peat-peat moss CM and treated with 1% WV, as detailed in Table 2. This variance in root morphology can be attributed to the soil’s heterogeneity concerning water and nutrient distribution, which significantly influences root system architecture. Soils present diverse environmental conditions that roots must adapt to for optimal growth and development. As Sinha et al. [19] elucidate, root systems dynamically adjust their architecture to efficiently exploit available resources, optimizing plant growth. The observed differences in root width and the root width/depth ratio highlight how specific treatments and culture mediums can impact root development, reflecting the roots’ adaptive strategies to varying environmental conditions. This adaptive capacity of root systems underscores the importance of selecting suitable culture mediums and treatments to enhance plant growth and development by facilitating optimal root system architecture.

### Root perimeter and root area

Seedlings cultivated in a date palm compost-vermicompost culture medium (CM) and treated with 1% WV showcased remarkable growth in root architecture, achieving the highest measurements in both root perimeter (7182.75) and root area (2145.97 cm²). This represents a substantial increase—99% in root perimeter and 87% in root area—compared to seedlings grown in a coco peat-peat moss CM and similarly treated with 1% WV, as outlined in Table 2. Furthermore, Fig. 1 elucidates significant positive correlations between root perimeter and total root length, as well as between root perimeter and root area, indicating that enhancements in one attribute beneficially impact others, promoting overall root system development. The augmentation in root growth observed with low concentrations of WV treatment could be attributed to the disruption of hormonal balances, particularly involving phenolic acids, as suggested by Lu et al. [14]. Such adjustments may enhance root elongation and expansion, thereby increasing the perimeter and area. Additionally, organic wastes, like those present in date palm compost, are known for their high apparent density, porosity, and water retention capacity, contributing positively to the CM’s quality [17]. The inclusion of date palm waste not only enhances the physical properties of the CM but also supports the growth and development of the root system by providing a conducive environment rich in nutrients and moisture. These findings align with those of Mohammadi Ghehsareh et al. [17], who noted the beneficial effects of various organic wastes on the quality of CMs, particularly when combined with other compost materials. The integration of date palm waste into the CM, especially when treated with specific concentrations of WV, evidently plays a pivotal role in optimizing root growth parameters. This synergy between organic waste components and wood vinegar indicates the potential for tailored CM formulations to significantly influence plant root development, enhancing the plant’s overall growth and productivity.

### Specific root length

The influence of CM, WV, and their interaction on specific root lengths was significant, as detailed in Table 1. Furthermore, Fig. 1 illustrates a significant and positive correlation between SRL and RP, indicating that changes in one could predict alterations in the other. Notably, seedlings treated with 1% WV and cultivated in the date palm compost-vermicompost CM exhibited the lowest SRL, which was reduced by 33% compared to seedlings treated with 0.5% WV and grown in the coco peat-peat moss CM, as reported in Table 2. This observed variation in SRL can be attributed to the biphasic dose-effect relationship of phenolic compounds present in WV, where lower concentrations stimulate growth and higher concentrations result in inhibition. Phenolic compounds, at lower concentrations, alongside low concentrations of acids and other allelochemical substances found in WV, are known to enhance the content of soluble sugar, induce protein expression, and increase root strength. These biochemical changes are conducive to seed germination and root growth, as discussed by Calabrese and Blain [20], Li et al. [21], and Wang et al. [22]. Such findings indicate the nuanced role of WV concentration in modulating root development, highlighting the importance of optimizing WV application to leverage its growth-stimulating effects while avoiding concentrations that may inhibit plant development. The study demonstrates the complex interactions between plant growth media, WV concentrations, and plant physiological responses, emphasizing the potential of carefully selected CM and WV treatments to enhance plant growth and development through the strategic manipulation of root architecture.

### Evaluation of growth parameters

#### Fresh and dry mass of root and shoot

The analysis underscored the significant impact of culture medium (CM), wood vinegar (WV), and their interplay on the fresh and dry mass of roots and shoots, as articulated in Tables 4 and 5. Specifically, seedlings grown in date palm compost-vermicompost CM, when treated with WV, demonstrated superior growth in terms of both fresh and dry mass compared to those in other CMs. The minimal fresh and dry mass were observed in seedlings treated with 0.5% WV within coco peat-peat moss CM, indicating the efficacy of the date palm compost-vermicompost CM in promoting plant growth. Furthermore, a direct and positive correlation was established between the fresh and dry mass of roots and shoots, indicating that enhancements in root biomass are accompanied by proportional increases in shoot biomass. The application of WV, particularly in the date palm compost-vermicompost CM, not only augmented the biomass but also favorably altered the shoot-to-root fresh mass ratio, highlighting the role of CM and WV concentration in optimizing plant growth dynamics. The growth-promoting effects of date palm compost are attributed to its enhancement of nutrient availability and efficiency, including both macro (N, P, K) and micro (Fe, Mn, Zn, Cu) nutrients, thereby facilitating increased growth and productivity. Similarly, WV has been recognized for its role as a catalyst in plant growth, activating enzymes and bolstering physiological and biochemical processes such as photosynthesis and nutrient absorption. Consistent with findings from Zhu et al. [23], the addition of WV and its constituents notably increased both the dry and fresh mass of plants, aligning with observations from the current study. Moreover, comparisons across different CM compositions revealed that specific ratios of peat moss, vermicompost, and palm peat within the CM significantly boosted plant biomass compared to alternative mixtures, as reported by Afsharipour et al. [24]. The inclusion of WV at optimal concentrations was found to enhance root growth, stress resistance, and anti-aging mechanisms, supporting the notion that WV and its components, including butanolide and furan derivatives, function as growth stimulants by mimicking hormonal activity or providing vital growth-promoting substances. The complexity of WV, characterized by its rich content of acids, phenols, vitamin B, and plant hormones such as karrikinolide, underpins its multifaceted role in enhancing plant growth. The beneficial impact of phenolic substances in WV, particularly at low concentrations, has been highlighted, showcasing their potential to significantly foster plant development. This comprehensive analysis reinforces the synergistic effect of selecting an appropriate CM in conjunction with WV treatment to optimize plant growth outcomes. The findings corroborate previous studies, suggesting that the strategic use of WV, alongside nutrient-rich CMs such as date palm compost-vermicompost, can significantly enhance the growth parameters of plants, setting a precedent for future agricultural practices and research.

**Table 4:** Mean comparison of culture medium (CM) and wood vinegar (WV) on growth parameters, RWC, and MSI of cucumber seedlings

Treatments			Fresh mass of							Dry mass of							
Culture medium			**WV (%)**			**Root (g)**		**Shoot (g)**		**shoot/root (g)**		**Root (g)**		**Shoot (g)**		**shoot/root (g)**	
coco peat – peat moss			untreated			2.38^c^		5.97^e^		2.52^f^		0.11^d^		0.58^d^		5.31^ef^	
			0.5			1.94^d^		5.85^e^		3.03^e^		0.10^d^		0.57^d^		5.63^de^	
			1			2.39^bc^		7.54^d^		3.15^e^		0.13^c^		0.66^c^		5.21^f^	
coco peat – vermicompost			0			2.48^abc^		10.51^b^		4.26^abc^		0.13^bc^		0.84^b^		6.44^ab^	
			0.5			2.55^abc^		9.83^c^		3.89^d^		0.14^ab^		0.84^b^		5.81^cd^	
			1			2.57^ab^		10.15^bc^		3.96^cd^		0.14^bc^		0.83^b^		5.88^cd^	
date palm compost -vermicompost			0			2.65^a^		10.54^b^		3.98^bcd^		0.14^bc^		0.94^a^		6.67^a^	
			0.5			2.63^a^		11.36^a^		4.32^ab^		0.15^a^		0.92^a^		5.95^cd^	
			1			2.49^abc^		11.05^a^		4.44^a^		0.15^a^		0.93^a^		6.13^bc^	
Culture medium		**WV (%)**			**Root length (cm)**		**Shoot length (cm)**		**Stem diameter (mm)**		**Stem sturdiness quotient**		**Leaf area (cm)**		**RWC (%)**		**MSI (%)**
coco peat – peat moss		untreated			12.64^b^		14.64^g^		4.33^b^		3.39^e^		27.16^e^		61.23^e^		65.99^e^
		0.5			11.06^c^		15.97^f^		4.44^b^		3.61^d^		28.95^e^		68.23^d^		73.01^abc^
		1			14.46^a^		16.89^f^		5.15 ^a^		3.28^e^		28.57^e^		71.75^d^		71.94^a^
coco peat – vermicompost		untreated			8.86^e^		20.57^c^		5.44 ^a^		3.78^c^		37.76^c^		84.30^ab^		73.02 ^ab^
		0.5			9.59^de^		18.01^e^		5.43 ^a^		3.32^e^		35.57^d^		86.49^a^		70.13^cd^
		1			10.52^cd^		19.60^d^		5.04 ^a^		3.90^bc^		38.12^c^		83.81^ab^		74.55^a^
date palm compost -vermicompost		untreated			11.52^c^		24.08^a^		5.35 ^a^		4.51^a^		42.38^b^		78.95^bc^		68.99^bcd^
		0.5			9.26^de^		24.19^a^		5.32 ^a^		4.55^a^		43.63^a^		77.74^c^		70.16^cd^
		1			9.06^de^		22.91^b^		5.53^a^		4.15^b^		43.94^a^		79.27^bc^		68.79^d^

Different letters in a column indicate significant differences at *p* ≤ 0.05 among the growing mediums, based on Duncan’s multiple-range test

**Table 5:** Variance analysis of the effect of the culture media (CM) and wood vinegar (WV) on photosynthesis pigments, chlorophyll fluorescence, and auxin of cucumber seedling

Variation sources	DF	SPAD	Chl a	Chl b	Chl a/b	Total Chl	CARs	F_v_/F_m_	Auxin
CM	2	349.25^**^	0.95^**^	0.059^**^	0.25^**^	1.46^**^	0.08^**^	0.095^**^	548.46^**^
WV	2	20.51^**^	0.008^**^	0.002^*^	50.24^**^	0.013^*^	0.002^*^	0.004^*^	126.23^**^
CM×WV	4	7.200^*^	0.65^**^	0.006^**^	0.097^*^	0.112^**^	0.012^**^	0.010^**^	104.75^**^
C.V.	-	3.73	3.61	7.85	4.31	4.28	5.55	4.21	10.19

* and ** indicate significant differences at *p* ≤ 0.05 and *p* ≤ 0.01 respectively based on Duncan’s multiple range test. Chl *a;* Chlorophyll *a*, Chl *b;* Chlorophyll *b*, Total Chl; total chlorophyll and CARs; carotenoids, fv/fm; photochemical efficiency of PSII

### Root length and shoot length

**Fig. 2:**
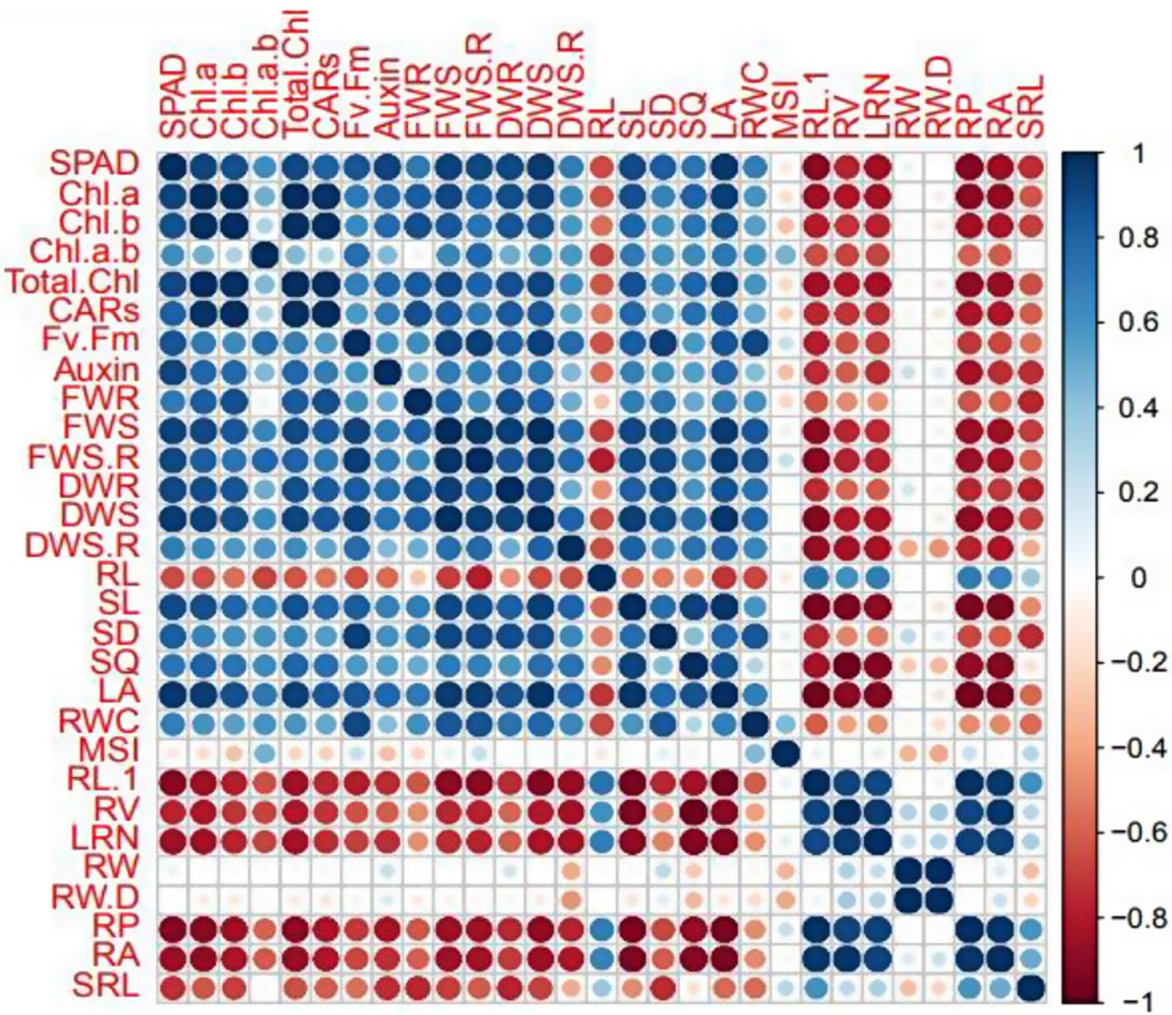
Pearson correlation analysis between evaluated traits in *Cucumis sativus* seedlings grown on the different culture media and treated with pistachio wood vinegar. SPAD, Greenness index; Chl.*a*, chlorophyll *a*; Chl.b chlorophyll *b*; Chl.*a.b*, chlorophyll *a/b*; Total.Chl, total chlorophyll; CARs, carotenoids; Fv.Fm, photochemical efficiency of PSII; FWR, fresh mass root; FWS, fresh mass shoot; FWS.R, ratio of fresh mass shoot to root; DWR, dry mass root; DWS, dry mass shoot; DWS.R, ratio of dry mass shoot to root; RL.1, total root length, SL, shoot length; SD, stem diameter; SQ, stem sturdiness quotient; SL, shoot length; PRL, particular root length, RV, root volume; LRN, lateral roots number; RW, root width; RW.D, root width to root depth; RP, root perimeter; RA, root area; SRL, specific root length

The study’s findings highlight the significant influence of CM, WV, and their interaction on the growth metrics of roots and shoots. Specifically, seedlings grown in coco peat-vermicompost CM without WV treatment exhibited the shortest root length (8.86 cm), whereas those treated with 1% WV in a coco peat-peat moss CM demonstrated the greatest root extension (14.46 cm), as evidenced in Table 4 and depicted in Fig. 2. Moreover, the maximal shoot length (24.19 cm) was observed in seedlings cultivated in date palm compost-vermicompost CM and treated with 0.5% WV, marking a 65% increase over those grown in coco peat-peat moss CM without WV. An intriguing aspect of these findings is the observed significant negative correlation between shoot length (SL) and root length (RL), contrasted by a positive correlation between SL and stem sturdiness quotient (SQ), as shown in Fig. 1. These outcomes resonate with the work of Ghouili et al. [25], who found that compost application in barley notably augmented shoot length and dry biomass. Similarly, Radhouani et al. [26] reported that compost usage facilitated stem elongation and dry matter accumulation in shoots, underscoring the beneficial impact of organic amendments on plant growth. Additionally, Zhang et al. [27] demonstrated that seed priming with 0.5% WV enhanced drought resistance in rice seedlings, attributing the effect to organic compounds such as butanolide, acetic acid, and catechol. The research further identifies guaiacol, a predominant component in pistachio WV, known for its antioxidant properties and inhibitory effects against fungal growth. This highlights the multifaceted role of WV, not just in promoting plant growth but also in offering protective benefits against pathogens. The study shows the combined effect of nutrient-rich vermicompost and the bioactive compounds in WV on enhancing cucumber seedling growth, particularly when treated with 0.5% WV in a date palm compost-vermicompost CM. The slow release of active acids and phenols in WV, even at low concentrations, is pinpointed as a mechanism for improving nutrient availability and consequently, shoot and seedling growth. This is supported by Luo et al. [28], who noted the growth-enhancing effects of low-concentration WV on plant development. Furthermore, Siriwardena et al. [29] and Zhang et al. [27] reported significant increases in shoot and seedling lengths across various crops when treated with WV, reinforcing the notion of WV’s beneficial impact on plant growth.

### Stem diameter and stem sturdiness quotient

The study delineates that the interaction between CM, WV, and their combined application profoundly influences stem diameter and stem sturdiness quotient, with statistical significance observed at *p* ≤ 0.01. Seedlings cultivated in date palm compost-vermicompost CM and treated with 1% WV exhibited the greatest stem diameter, measuring 5.53 cm. Moreover, the stem sturdiness quotient, which is a measure of the stem’s robustness relative to its length, was significantly higher (38% increase) in seedlings grown in date palm compost-vermicompost CM and treated with 0.5% WV compared to those grown in coco peat-peat moss CM and treated with 1% WV, as highlighted in Table 4. On the other hand, Fig. 1 further substantiates these findings, illustrating a significant and positive correlation between stem diameter (SD) and shoot length (SL), as well as between stem sturdiness quotient (SQ) and shoot length (SL). This indicated that as the stem’s thickness and robustness increase, so does the shoot length, suggesting a harmonious growth pattern that benefits overall plant structure and stability. The study by Rahbarian and Sardoei [30], suggests that palm peat CM performs comparably to peat moss in supporting plant growth, indicating the potential of palm peat as a sustainable alternative to peat moss in horticultural practices. This is particularly relevant given the environmental concerns associated with the extraction of peat moss. Furthermore, our research shows the role of acidic substances in WV in facilitating intracellular acidification and enhancing cellular activity, thereby contributing to increased plant vigor. The positive impact of WV on plant growth is also attributed to its stimulation of hormonal (auxin and gibberellin) and enzyme activities, alongside enhancing nitrogen absorption, as demonstrated in studies on tomato plants by Zhu et al. [23].

### Leaf area

The investigation into the effects of CM, WV, and their synergistic interactions reveals a significant impact on leaf area, as indicated in Table 6. Notably, seedlings cultivated in date palm compost-vermicompost CM exhibited a pronounced increase in leaf area, which was further enhanced with higher concentrations of WV. Specifically, the application of 1% WV derived from pistachio to seedlings in date palm compost-vermicompost medium resulted in a 61% increase in leaf area compared to seedlings grown in coco peat-peat moss CM without WV treatment, as detailed in Table 4 and illustrated in Fig. 2. Further analysis, as shown in Fig. 1, established a significant and positive correlation between leaf area (LA) and both fresh mass of shoot (FMS) and dry mass of shoot (DMS). This relationship underscores the integral role of leaf area in the overall biomass and productivity of the plant, with larger leaves contributing to increased photosynthetic capacity and, consequently, greater biomass accumulation. The complex composition of WV, predominantly consisting of acids and phenols as identified in Table 3, is known for its high biological activity and growth-promoting properties. The findings of Zhu et al. [23], which demonstrate significant enhancements in plant height, total leaf number, green leaf number, leaf area, and effective branch number in rapeseed following treatment with a 400-fold dilution of WV, align closely with the outcomes of this study. Such results attest to the efficacy of WV in stimulating vegetative growth and development. Moreover, the influence of the cultivation medium’s composition on leaf area has been well-documented in the literature [31], highlighting the importance of selecting appropriate substrate materials and amendments for optimizing plant growth. The present study further corroborates the critical role of both CM and WV in enhancing leaf development, suggesting that their careful selection and application can significantly improve plant growth parameters, thereby potentially increasing yield and productivity in agricultural and horticultural practices.

**Table 6:** Mean comparison of the effect of the culture media (CM) and wood vinegar (WV) on photosynthesis pigments, chlorophyll fluorescence, and auxin of cucumber seedling

Treatments		SPAD	Chl *a* (mg/g FW)	Chl *b* (mg/g FW)	Chl *a*/*b* (mg/g FW)	Total Chl (mg/g FW)	CARs (mg/g FW)	F_v_/F_m_	Auxin (ppm/g fw)
CM	WV (%)								
**coco peat - peat moss**	**0**	26.87^d^	0.75^f^	0.25^b^	2.97^d^	1.01^f^	0.34^e^	0.50^e^	25.52^c^
	**0.5**	26.47^d^	0.50^h^	0.14^d^	3.66^ab^	0.64^h^	0.21^g^	0.62^d^	21.83^c^
	**1**	28.67^d^	0.59^g^	0.18^c^	3.36^c^	0.76^g^	0.26^f^	0.66^cd^	22.87^c^
**coco peat - vermicompost**	**0**	33.40^c^	0.90^e^	0.25^b^	3.60^abc^	1.16^e^	0.34^de^	0.76^ab^	26.25^c^
	**0.5**	35.33^c^	0.99^d^	0.29^b^	3.50^ab^	1.28^d^	0.37^cd^	0.79^ab^	31.97^b^
	**1**	34.83^c^	1.14^c^	0.32^a^	3.52^ab^	1.46^c^	0.43^b^	0.72^bc^	32.11^b^
**date palm compost -vermicompost**	**0**	38^b^	1.18^c^	0.33^a^	3.55^abc^	1.52^c^	0.42^c^	0.80^a^	32.50^b^
	**0.5**	37.97^b^	1.37^a^	0.36^a^	3.78^a^	1.73^a^	0.49^a^	0.76^ab^	34.09^b^
	**1**	43.25^a^	1.26^b^	0.34^a^	3.71^bc^	1.60^b^	0.42^b^	0.79^a^	50.31^a^

Different letters in a column indicate significant differences at *p* ≤ 0.05 among the growing mediums, based on Duncan’s multiple range test. Chl *a;* Chlorophyll *a*, Chl *b;* Chlorophyll *b*, Total Chl; total chlorophyll and CARs; carotenoids, F_v_/F_m_; photochemical efficiency of PSII

### Evaluation of RWC and MSI

Our study’s findings highlight the significant impact of CM and WV treatments, and their interactions, on the RWC and MSI of seedlings, as detailed in Table 6. Notably, seedlings cultivated in coco peat-peat moss CM without WV treatment exhibited the lowest RWC (61.23%) and MSI (65.99%). In contrast, the application of WV (0.5% and 1%) in a coco peat-vermicompost CM significantly enhanced both RWC and MSI, recording the highest values at 86.49% and 74.55%, respectively. These improvements represent a 41% increase in RWC and a 13% increase in MSI compared to seedlings grown in coco peat-peat moss CM without WV treatment, as shown in Table 4. These results underscore the beneficial effects of selecting an appropriate CM and employing WV treatments on enhancing plant water retention and membrane stability, crucial factors for plant stress resistance and overall vitality. The findings align with research by Benabderrahim et al. [32], which demonstrated that the application of date palm waste compost not only increased the biomass of alfalfa but also maintained a high RWC, indicating the compost’s role in improving water use efficiency and stress tolerance in plants. Our study’s outcomes suggest that integrating WV, particularly at specific concentrations, with nutrient-rich CMs like coco peat-vermicompost can significantly improve plant physiological parameters such as RWC and MSI. These enhancements contribute to better water management within the plant, increased resistance to environmental stresses, and potentially improved growth and yield, highlighting the importance of optimizing CM and WV treatments for agricultural and horticultural practices.

### Evaluation of photosynthetic pigments and chlorophyll fluorescence

Our study reveals that the CM, WV, and their interaction significantly influence the SPAD values, chlorophyll *a*, *b*, *a*/*b* ratio, total chlorophyll, and carotenoids, as indicated in Table 5. Notably, the photosynthetic pigments in seedlings grown in the date palm compost-vermicompost CM were higher than in other CMs. Specifically, the SPAD values, which indicate chlorophyll content, showed a significant increase of 61% in seedlings grown in coco peat-peat moss CM and 14% in those grown in date palm compost-vermicompost CM, both treated with 1% WV, compared to seedlings in the latter CM untreated with WV, as shown in Table 7. Furthermore, Fig. 1 highlights a positive correlation between SPAD values and chlorophyll *a* (Chl.*a*) and chlorophyll *b* (Chl.b). The highest levels of chlorophyll *a*, *b*, total chlorophyll, and carotenoids were observed in seedlings treated with 0.5% WV in the date palm compost-vermicompost CM, with these pigments increasing by 2.3, 2.7, 2.6, and 2.7 times, respectively, compared to those treated with the same concentration of WV but grown in coco peat-peat moss CM. Additionally, the chlorophyll *a*/*b* ratio in seedlings grown in date palm compost-vermicompost CM and treated with 0.5% WV increased by 21% compared to those untreated with WV and grown in coco peat-peat moss CM. The study also identified a significant correlation between total chlorophyll and its constituents (Chl.*a*, Chl.*b*, and carotenoids), underlining the interdependence of these pigments in photosynthesis, which reflects the physiological state and vitality of the plant. Wood vinegar treatment, as concluded by Theerakulpisut et al. [33], modulates physiological processes that mitigate membrane damage, enhance ionic homeostasis, and prevent chlorophyll degradation. Salehi Sardoui et al. [34] found that the highest chlorophyll index occurred in substrates made from waste palm trees, whereas coco chip substrates showed the lowest due to their water-holding capacity and lower content of certain elements critical for photosynthesis. The date palm compost-vermicompost CM, having higher nitrogen content, suggests nitrogen’s crucial role in chlorophyll structure and function. Moreover, the F_v_/F_m_ ratio, a measure of Photosystem II (PSII) efficiency, increased significantly in seedlings grown in the date palm compost-vermicompost CM, whether treated with 1% WV or untreated, compared to those untreated with WV and grown in coco peat-peat moss CM (Table 7). This ratio is essential for assessing the efficiency of PSII, with decreases under stress indicating reduced PSII efficiency. The beneficial properties of vermicompost, such as enhanced water retention and nutrient absorption capacities, contribute to the stability of the photosynthetic apparatus, improving plant resilience and growth.

**Table 7:** Chemical properties of the materials in culture mediums used in this study

Culture media	EC (mS/cm)	pH	*N* (%)	K (%)	*P* (%)	Mn (ppm)	Cu (ppm)	Fe (ppm)	Zn (ppm)
Peat moss	0.754	6.73	0.11	1.26	0.86	0.20	0.16	0.30	0.066
Vermicompost	2.66	6.58	0.16	1.33	0.44	0.36	0.13	0.66	0.074
Date palm compost	1.73	6.78	0.12	0.86	0.39	0.22	0.18	0.33	0.029
Coco peat	1.10	6.92	0.10	0.44	0.26	0.16	0.11	0.31	0.020

### Evaluation of auxin content in seedlings

Our study’s results indicate a significant effect of the CM, WV, and their interaction on auxin content, as detailed in Table 5. Auxin levels were notably higher in seedlings grown in date palm compost-vermicompost CMs, with subsequent decreases observed in those grown in coco peat-vermicompost and coco peat-peat moss CMs, respectively. The peak auxin concentration was found in seedlings cultivated in the date palm compost-vermicompost CM and treated with 1% WV, showcasing a 130% increase compared to seedlings grown in coco peat-peat moss CM and treated with 0.5% WV, as reported in Table 7. In addition, Fig. 1 reveals a positive and substantial correlation between auxin levels and total chlorophyll content. Auxin, a pivotal plant growth regulator synthesized from the amino acid tryptophan, is instrumental in controlling cell elongation, division, and differentiation, with its accumulation influenced by various factors including organic compounds, the presence of Karrikins in WV, and the nutrients available in vermicompost and date palm compost CMs. Karrikins, identified in WV, may interact with endogenous plant hormones to regulate growth processes such as hypocotyl elongation, thereby enhancing seedling length, as suggested by Meng et al. [35]. The observed higher auxin content in seedlings grown in date palm compost-vermicompost CMs can be attributed to the elevated nitrogen levels in these mediums, essential for auxin biosynthesis. This relationship indicates the crucial role of nitrogen not only in auxin construction but also in facilitating greater nitrogen uptake from such enriched CMs, contributing to enhanced seedling growth. Furthermore, the interaction between auxin and nitrogen highlights a synergistic mechanism where both function as signaling molecules to stimulate root development and nutrient absorption, as discussed by Marchive et al. [36]. This dynamic shows the complexity of plant growth regulation, where hormonal balance and nutrient availability interplay to optimize growth and development. The findings elucidate the significant impact of CM composition and WV treatment on hormonal regulation within plants, particularly concerning auxin levels, which subsequently influence overall plant growth and photosynthetic efficiency. This insight emphasizes the potential benefits of integrating organic amendments and bioactive compounds in agricultural practices to enhance plant health and productivity.

## Conclusions

In our study, the comprehensive analysis of the effects of CM, WV, and their interaction on various plant growth parameters elucidates several key findings. First, the combination of date palm compost-vermicompost CM and WV treatment significantly enhanced root and shoot growth, leaf area, photosynthetic pigment concentration, and plant hormonal balance, particularly auxin levels. This positive impact was attributed to the nutrient-rich composition of the CM and the bioactive components of WV, which together promote cellular activity, improve water and nutrient uptake efficiency, and stimulate photosynthesis and growth hormone synthesis. The study revealed a notable increase in root and shoot biomass, chlorophyll content, and auxin concentration, especially in seedlings treated with specific concentrations of WV in date palm compost-vermicompost CM, indicating the synergistic potential of organic amendments and bioactive treatments in agricultural practices. Furthermore, our research underscored the importance of optimizing WV concentration and selecting suitable CMs to achieve the best growth outcomes, highlighting the role of WV in enhancing drought resistance and photosynthetic efficiency through the modulation of physiological and biochemical processes. Our study also pointed to the beneficial effects of vermicompost and date palm compost in improving the physical and chemical properties of the growing medium, thereby facilitating better plant development. In general, our findings indicated that the application of 0.5% WV under date palm compost-vermicompost CM improved the seedling’s quality of cucumber compared to the other treatments. To sum up, the integration of wood vinegar and nutrient-rich culture mediums like date palm compost-vermicompost offers a promising avenue for enhancing plant growth, stress resistance, and photosynthetic efficiency. Future research should explore the mechanistic pathways through which WV and compost amendments exert their growth-promoting effects, with a particular focus on optimizing application rates and combinations to maximize agricultural productivity and sustainability.

## Materials and methods

### Experimental site, and treatments

This study was carried out at Jiroft University’s research greenhouse in 2022, under controlled environmental conditions including an average day/night temperature of 30/25 ± 5 °C, relative humidity of 70 ± 5%, and a light intensity of approximately 240 µmol m^− 2^ S^− 1^. The experimental setup was designed as a factorial experiment within a randomized complete block design (RCBD) with five replicantions, featuring three replications to assess the effects of different culture media (CMs) and concentrations of pistachio wood vinegar (WV) on cucumber seedling growth. The culture media used were divided into three combinations based on a volume-to-volume (v/v) ratio: coco peat-peat moss (25:75), coco peat-vermicompost (25:75), and date palm compost-vermicompost (75:25). This specific ratio configuration was selected based on preliminary tests that demonstrated optimal adhesive strength and water-holding capacity for seedling development. Peat moss, vermicompost, date palm compost, and coco peat, were supplied from companies of golfgreen peat moss, vermino vermicompost, Golsehreh, and prococo companies, respectively. The chemical properties of each of these components of the culture medium are presented in Table 8. The study also incorporated three levels of pistachio WV concentration (0, 0.5, and 1%) after pH normalization with KOH to reach pH = 8.2, with the control seedlings being treated with distilled water to represent the 0% WV concentration. Throughout the growth period, the plants received a weekly feeding of half-strength Hoagland’s nutrient solution. To mitigate potential damage from whiteflies, a common pest in greenhouse environments, the plants were housed within insect-netted chambers. The seedlings underwent WV treatment a total of four times. At the 30-day mark, coinciding with the stage of three fully expanded leaves, the seedlings were carefully extracted from the pots. Subsequently, a separation process was conducted where the shoots were detached from the roots post-washing, preparing them for further analysis.

**Table 8:** The main polar component of pistachio wood vinegar

Retention time (min)	Compounds	Formula	Relative content (%)	Molar mass (g mol^− 1^)
2.034	Bis (trimethylsiloxy) methylsilane	C_7_H_22_O_2_Si_3_	7.785	222
5.046	1-Hydroxy-2-pentanone	C_5_H_10_O_2_	3.448	102
2.092	2,4,6-Cycloheptatrien-1-one, 3,5-bis-trimethylsilyl	C_13_H_22_OSi_2_	7.316	250
5.721	2,3-dimethyl-2-cyclopentenone	C_7_H_10_O	3.503	110
5.442	3-Methyl-2-hydroxy-2-cyclopentenone	C_6_H_8_O_2_	3.635	112
4.282	2-Methyl-2-cyclopenten-1-one	C_6_H_8_O	5.034	96
6.059	2-Myristynoyl-glycinamide	C_16_H_28_N_2_O_2_	2.077	280
2.481	3-Cyclopentene-1-acetaldehyde, 2-oxo^−^	C_7_H_8_O_2_	8.221	124
9.384	2-Methoxy-4-methyl phenol	C_8_H_10_O_2_	2.685	138
1.832	Hexamethylcyclotrisiloxane	C_6_H_18_O_3_Si_3_	1.493	222
3.413	1-(2-furanyl)- Ethanone	C_6_H_6_O_2_	5.721	110
4.541	Phenol	C_6_H_6_O	6.350	94
13.446	2,6-Dimethoxyphenol	C_8_H_10_O_3_	6.367	154
6.852	Guaiacol	C_7_H_8_O_2_	13.336	124
6.542	p-Octylphenol	C_14_H_22_O	3.195	206

### Date palm composting

In the process of preparing compost from date palm trunks, the initial step involved cutting the trunks into pieces measuring 3 × 15 mm, followed by a leaching process aimed at reducing salinity levels. Subsequently, these prepared pieces were organized into rows measuring 20 m in length and 1.5 m in width, where moisture was meticulously supplied via tape placed along each row. To enhance the microbial activity and expedite the decomposition of organic material, approximately 23 kg of pure nitrogen, alongside 2000 ppm of the fungicide Mancozeb, were evenly distributed across the layers of the compost pile. To regulate temperature and ensure adequate oxygen supply, the materials within the compost were systematically turned every two days. After 40 days, a second leaching was conducted to further diminish the salinity of the compost. This comprehensive composting procedure spanned 4 to 5 months, culminating in the production of compost characterized by its dark brown to black hue and a distinctive wet soil aroma [24].

### Preparation of wood vinegar

The tree pistachio was selected in this study. Pyroligneous acid was extracted following the procedure described by Bouket et al. [37]

### GC-MS analysis of wood vinegar

The principal polar component of wood vinegar was determined utilizing a gas chromatography-mass spectrometer (GC-MS) system (Agilent Technologies 7890B, USA). Pure helium served as the carrier gas, maintaining a constant flow rate of 1 mL per minute. A ms-DB capillary column was employed for the separation of components. The operational temperature protocol for the column started at 60 °C and was increased to 250 °C at a rate of 5 °C per minute, where it was then held steady for 2 min. The identification of polar compounds was achieved through the analysis of retention times and the comparison of mass spectrometry data against entries in the mass spectral library provided by NIST. The quantification of these compounds was based on the relative peak area method, as outlined by Ma et al. [38]. Table 3 lists the main polar component identified in pistachio wood vinegar.

#### Measurement of root architecture

Upon concluding the experiment, root imagery was subjected to analysis via the GiA Roots software, which facilitated the conversion of these images into quantifiable data. This analysis aimed to evaluate root growth rates by examining the architectural characteristics of the roots. The attributes measured encompassed a comprehensive array of parameters: total root length, root volume, the number of lateral roots, root width, and the ratio of root width to depth. Additional characteristics included root perimeter, root area, specific root length, the maximum number of roots per plant, median number of roots, root bushiness (a measure of root branching density), root depth, root surface area, root convex area (indicating the outer boundary of the root system), root solidity (reflecting the compactness of the root structure), average root width diameter, ellipse aspect ratio (describing the shape of the root system in an elliptical representation), and the minor axis of the ellipse. These parameters collectively provided a detailed assessment of the root system’s growth and development [39].

### Measurement of growth parameters

The fresh mass of both shoots and roots was precisely measured using a digital scale with an accuracy of ± 0.001 g. Following the weighing process, the samples were subjected to a drying procedure in an oven, maintained at a temperature of 70 °C, for 48 h to achieve a constant dry mass [40]. The diameter of the stems was determined using a digital caliper, ensuring accurate measurements. For the assessment of leaf area, total root length, and stem length, the Digimizer software, version 5.4.9, was employed. This software facilitates the accurate digital analysis of these parameters. Additionally, the concept of stem sturdiness quotient (SSQ), which is the ratio of stem height to stem diameter, was calculated using a specific equation, referred to as Eq. (1). This quotient serves as an indicator of the structural integrity and robustness of the stem, providing insights into the plant’s ability to support its biomass. The methodology for calculating the SSQ and its significance in plant physiology was detailed by Bayala et al. [41].

1 $$SSQ=\frac{\text{s}\text{t}\text{e}\text{m} \text{h}\text{e}\text{i}\text{g}\text{h}\text{t}}{\text{s}\text{t}\text{e}\text{m} \text{d}\text{i}\text{a}\text{m}\text{e}\text{t}\text{e}\text{r}}$$

### Relative water content (RWC) and membrane stability index (MSI)

To assess the Relative Water Content (RWC) of plants, the third fully developed leaf from each plant was selected for sampling. The initial step in this procedure involved measuring the fresh weight (FW) of these leaves immediately after their collection. Following this, the leaves were placed in test tubes filled with distilled water and stored at 4 ºC for 24 h, a step aimed at allowing the leaves to reach full turgidity. Upon completion of this period, the leaves were removed from the water and gently blotted to remove excess surface moisture, after which their turgid weight (TW) was recorded. Subsequently, to ascertain the dry weight (DW) of the leaves, they were subjected to a drying process in an oven set at a temperature of 70 ºC for 24 h. This ensured the removal of all moisture content, providing a basis for calculating the dry weight of the leaves. The calculation of RWC was then conducted based on a specific formula, referred to as Eq. (2). This formula, as outlined by Ritchie et al. [42], facilitates the determination of the proportion of water content relative to the potential water content that the leaf can hold, offering insights into the plant’s hydration status and its ability to maintain water under varying environmental conditions.

2 $$RWC=\frac{\text{F}\text{W}-\text{D}\text{W}}{\text{T}\text{W}-\text{D}\text{W}}\times 100$$

To evaluate the Membrane Stability Index (MSI), 0.1 g of tissue samples, specifically obtained from the third leaf, was immersed in test tubes containing 10 ml of double distilled water. These test tubes were then placed in a Bain-Marie, a type of water bath, set at a temperature of 40 °C for 10 min. Following this initial treatment, the electrical conductivity of the water containing the leaf samples (EC1) was measured to assess the initial leakage of ions from the cells, indicative of membrane integrity under a relatively mild temperature stress. Subsequently, to further evaluate membrane stability under more extreme conditions, the same samples were subjected to a higher temperature treatment in the Bain-Marie, this time set at 100 °C for 30 min. This step aimed to induce further ion leakage from the damaged cells, reflecting the extent of membrane disruption. After allowing the samples to cool down to room temperature, the electrical conductivity of the water (EC2) was measured again. The Membrane Stability Index (MSI) was then calculated using a specific formula, denoted as Eq. (3). This equation considers the difference in electrical conductivity measurements before and after the high-temperature treatment to provide a quantitative measure of membrane stability. Higher MSI values are indicative of greater membrane integrity and stability, suggesting better tolerance to temperature-induced stress. This method is described by Sairam and Srivastava [43].

3 $$MSI=1-\frac{{EC}_{1}}{{EC}_{2}}\times 100$$

#### Measurement of biochemical characteristics

Biochemical analyses were conducted on fully developed third leaves to assess various parameters crucial for understanding plant physiological status. The greenness index, an indicator of chlorophyll content, was measured using a hand-held chlorophyll meter (SPAD Model 505), which provides a non-destructive method of assessing chlorophyll levels directly from the leaf surface. For more detailed chlorophyll and carotenoid quantification, a spectrophotometer (UV/VIS, Perkin Elmer, USA) was employed. This analysis was performed at specific wavelengths of 470 nm for carotenoids, 663 nm for chlorophyll a, and 645 nm for chlorophyll b, adhering to the methodology established by Lichtenthaler and Buschmann [44]. Chlorophyll fluorescence parameters, indicative of the photosynthetic efficiency and health of the photosystem II (PSII), were measured using a portable chlorophyll fluorometer (Hansatech Instruments Ltd., UK). The protocol involved dark-adapting the second expanded leaf for 20 min using special clamps to ensure the absence of light-induced effects on fluorescence measurements. The device’s sensor then emitted light at a wavelength of 695 nm onto the leaf, capturing the photochemical quantum yield of PSII (F_v_/F_m_) by recording the minimal fluorescence (F_0_) in dark-adapted conditions and maximal fluorescence (F_m_) upon light adaptation. The maximal variable fluorescence (F_v_) and the efficiency of PSII (F_v_/F_m_) were calculated based on these measurements, following the method outlined by Genty et al. [45]. Additionally, the concentration of auxin, a crucial plant hormone involved in growth regulation, was determined using a microplate reader (Epoch Biotech, USA). This quantification was performed according to the protocol described by Lwin et al. [46].

### Data analysis

For the statistical analysis of the data collected in the study, SAS software, version 9.4, served as the primary tool. To determine the significance of differences between the means of various treatments, Duncan’s multiple range test was employed, with a significance level set at *P* ≤ 0.05. In addition to SAS, the R software was utilized for the calculation of correlation coefficients between the studied traits.

## Data Availability

The data used in this study is openly available, and the data used is available upon request from the corresponding authors A.S. and A.M.
